# Quantitative LAMP and PCR Detection of *Salmonella* in Chicken Samples Collected from Local Markets around Pathum Thani Province, Thailand

**DOI:** 10.1155/2020/8833173

**Published:** 2020-07-09

**Authors:** Virun Vichaibun, Panan Kanchanaphum

**Affiliations:** Biochemistry Unit, Department of Biomedical Science, Faculty of Science, Rangsit University, Pathum Thani 12000, Thailand

## Abstract

*Salmonella* is a bacterium that infects people when they consume contaminated food or liquids. To prevent humans from becoming ill, it is useful to have an efficient method of detecting *Salmonella* before the disease is passed on through the food chain. In this research, the efficiency of *Salmonella* detection was compared using the following four methods: conventional loop-mediated isothermal amplification (LAMP), PCR, quantitative LAMP (qLAMP), and qPCR. The artificial infection of chicken samples started with incubating of 10 mL of 10^8^ CFU of *S. typhimurium* for 6 hr. and enriching for 2 hr. to represent real contamination of the samples. The results show that the sensitivity of *Salmonella* DNA detection in PCR, qPCR, LAMP, and qLAMP were 50 ng, 5 ng, 50 pg, and and 500 fg, respectively. Thirty samples of 10 g chicken were collected from 10 markets in Pathum Thani, Thailand; then, the infection was detected. The conventional LAMP, qLAMP, and qPCR methods detected *Salmonella* in all the chicken samples. However, the conventional PCR method detected *Salmonella* infection in only eight of the samples. Overall, the qLAMP method had the highest sensitivity of *Salmonella* DNA detection.

## 1. Introduction

Salmonellosis is a major foodborne infectious disease occurring worldwide, which is caused by the Gram-negative zoonotic pathogen, Salmonella [1]. Salmonella is a member of the Enterobacteriaceae family, which has been divided into the following two species: *S*. *enterica* and *S*. *bongori. S*. *enterica* has been further divided into six subspecies that include over 2,500 serotypes [2]. Almost all *Salmonella* outbreaks have been broadly ascribed across multiple food categories, and many people have become infected by consuming contaminated food originating from animals (such as eggs, beef, poultry, and milk) [3]. The symptoms of patients diagnosed with typhoid fever, caused by *Salmonella enterica serovar Typhimurium*, are similar to other illnesses such as Leptospirosis and *Streptococcus pneumoniae* infection [4]. Powerful and more efficient methods of detecting *Salmonella* are still required.

The conventional method of detecting and identifying *Salmonella* is a microbiological procedure, which requires multiple subculture steps followed by biochemical and serological testing. The method is time consuming (5 to 7 days) and very labor intensive. Molecular methods such as PCR and quantitative PCR (qPCR) [5] have been employed in *Salmonella* detection due to their specificity, sensitivity, and rapidity, [6–8]. Many reports showed Salmonella contamination in raw food especially in chicken was detected by PCR [9, 10]. However, the PCR and qPCR methods require sophisticated instruments and are time consuming. Fortunately, an innovative technique has been developed to negate these issues.

Loop-mediated isothermal amplification (LAMP) is a method of amplifying DNA under isothermal conditions. LAMP has been used in place of PCR because it provides high specificity, sensitivity, and rapidity. PCR consists of four specific primers based on six specific sequences applied to the DNA target and takes about one hour for the reaction time at 60°C-65°C for 45-60 min [11]. The method is more practical for microorganism detection because complicated instruments are unnecessary. Many publications in food and nutrition reported for the LAMP application such as [12] detected pork meat contamination in Halal food using LAMP.

The aim of this research is to compare the efficiency of LAMP, PCR, qualitative PCR (qPCR), and qualitative LAMP (qLAMP) by detecting *Salmonella* on chicken samples collected from markets around Pathum Thani province in Thailand.

## 2. Materials and Methods

### 2.1. Bacterial Strain and Culture Conditions

*Salmonella enterica* subsp. *enterica* serovar Typhimurium ATCC23566 was used in this study. The non-Salmonella strains belonged to the *Escherichia*, *Bacillus*, *Klebsill*a, *Shigella*, and *Enterobacter* genera. All the bacterial cultures were obtained from the Department of Microbiology, Faculty of Science, Rangsit University. For the cultivation, *Salmonella* was subcultured on xylose lysine deoxycholate agar media (XLD; Merck) and incubated at 37°C overnight (16 hr). The non-Salmonella strains were sub cultured on Luria-Bertani agar plate (10 g tryptone; 5 g yeast extract, 10 g NaCl; 15 g agar; and H_2_O to 1,000 mL) and incubated at 37°C overnight. The culture was used for DNA extraction using the GF-1 Bacterial DNA extraction kit (Vivantis, Malaysia) and measuring the concentration of DNA by using NanoDrop 2000c Spectophotometer (Thermo Scientific).

### 2.2. Primer Design for LAMP and PCR

A set of two pairs of primers, comprising of two inner primers (FIP and BIP) and two outer primers (F3 and B3) that could recognize six sequences of the *Salmonella* invasion gene (*invA*) were designed for the LAMP reaction (GenBank Accession Number M90846) [13]. To compare the efficiency of the detection methods, the F3 and B3 outer primers were used for the PCR. The nucleotide sequences of each primer are shown in Table 1.

### 2.3. LAMP and qLAMP Reaction

All the LAMP reactions were performed in 25 *μ*L of 1x *Bst* DNA polymerase buffer containing 5 mM MgSO_4_, 400 mM betaine (Sigma), 1.2 mM dNTPs, 0.8 *μ*M F3 and B3 primers, 2 *μ*M FIP and BIP primers, 8 U *Bst* DNA polymerase (New England Biolabs), and 10 ng of each DNA extract as a template. The reactions were incubated at 65°C for 45 min, which was followed by enzyme inactivation at 80°C for 5 min. qLAMP amplification was performed by adding 0.5 *μ*L of SYBR green I dye (Invitrogen, Carlsbad, CA) to the normal LAMP reaction.

### 2.4. PCR and qPCR Reactions

To compare the detection efficiency, a PCR assay targeting the *Salmonella invA* gene was performed in parallel with LAMP primer, as shown in Table 1. In addition, SYBR green I dye was used to enhance the specificity in the qPCR reaction. The PCR amplification reaction contained 1x *Taq* DNA polymerase buffer, 1.2 mM dNTPs, 0.8 *μ*M F3 and B3 primers and 8 U *Taq* DNA polymerase (New England Biolabs), and 5 ng of each DNA extract as a template in a final volume of 25 *μ*L. The cycling conditions comprised of a single initial denaturation at 94°C for 2 min followed by 30 cycles at 90°C for 30 sec (denaturation), 60°C for 30 sec (annealing), 72°C for 30 sec (extension), and a final extension step at 72°C for 5 min. After the PCR amplification, the products were analyzed by electrophoresis using 1.5% agarose gel, which was stained with ethidium bromide. Then, the gels were visualized under ultraviolet light.

### 2.5. The Specificity and Sensitivity of the LAMP, PCR, qLAMP, and qPCR Reactions

For the specificity testing, 500 ng/*μ*L DNA templates of *Salmonella typhimurium* and non-Salmonella strains were subjected to all four assaysใ

The DNA sensitivity testing for all the bacterial strains was 10-folds serial dilution from 5 *μ*g/*μ*L to 500 fg/*μ*L for all four methods. The sensitivity test was triple duplication experiment.

### 2.6. Artificial Contamination of the Chicken Samples with *Salmonella*

For the experiment, uncooked chicken was collected from markets around Pathum Thani province in Thailand. Initially, 10 g of chicken breast samples was transferred to a sterile container and washed twice with 10 mL of sterile distilled water then washed once with 5% trisodium phosphate (to eliminate the background flora). Next, the samples were rinsed with 10 mL of sterile distilled water. After that, they were dried inside the hood under ultraviolet light for 3 min [14]. Then, the prepared samples were incubated in 10 mL of 10^8^ CFU of *S*. *typhi* for 6 hr. and 1.5 mL of inoculated food was sampled at 0, 1, 2, 4, and 6 hr. time points. The solution was left to stand for 10 min to allow the particulate matter to settle at the bottom. Subsequently, the upper portion was collected and centrifuged at 10,000 g for 10 min. The DNA was extracted from the pellet by following a simple boiling method [15]. Finally, the extracted DNA was ready to be used in the amplification.

### 2.7. The Detection of *Salmonella* in Chicken Samples Gathered from Local Markets

Thirty chicken samples (chicken breast) were collected from 10 local markets (three samples from one market) in Pathum Thani province, Thailand. All the samples were examined immediately after purchase then sent to a laboratory in an ice box. The DNA extracted from the samples (10 g) was used in the LAMP and PCR detection. The bacterial strains were incubated in LB (Luria-Bertani) agar plate at 37°C for 18 hr. Colonies were taken by sterile loopful and incubated in LB broth at 37°C for 18 hr. Then, such culture grown in LB broth was taken for the experiment.

## 3. Results

### 3.1. Specificity Test of LAMP and PCR

**Figure 1:**
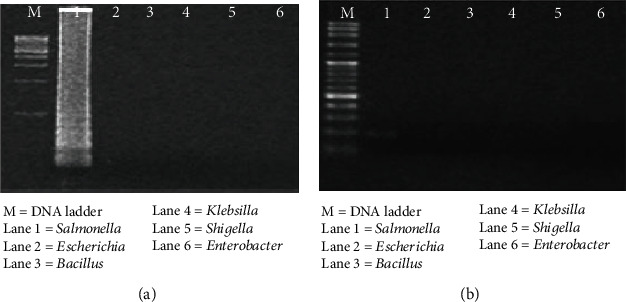
The specificity test of LAMP and PCR technique. (a) The LAMP. (b) The PCR method. M: DNA marker; 1: *Salmonella*; 2: *Escherichia*; 3: *Bacillus*; 4: *Klebsilla*; 5: *Shigella*; and 6: *Enterobacter.*

This results showed that the set of LAMP and PCR primers were specific-only Salmonella culture. The LAMP product and 198 bp PCR product appeared in lane 1 shown in Figures 1(a) and 1(b).

### 3.2. Sensitivity Test of LAMP, PCR, qLAMP, and qPCR

**Figure 2:**
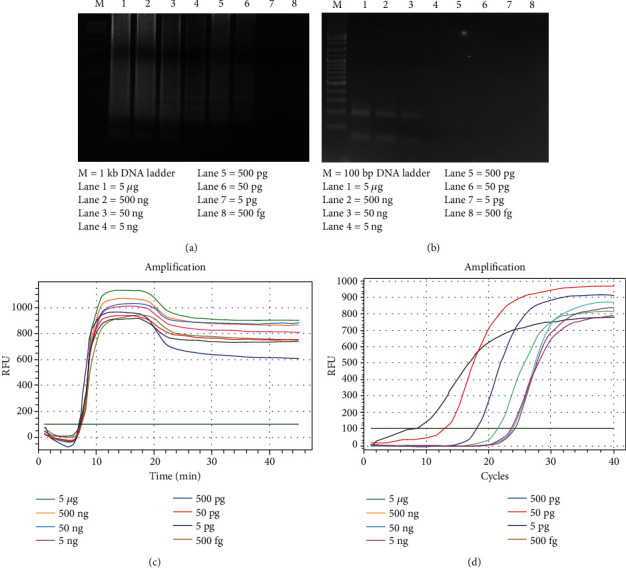
The sensitivity of all four methods in pure *Salmonella* culture by LAMP, PCR, qLAMP, and qPCR. (a) The LAMP method. M: 1 kb DNA marker; 1-8: 10-fold serial dilution of Salmonella DNA from 5 *μ*g–500 fg. (b) The PCR method. M: 100 bp DNA marker; 1-8: 10-fold serial dilution of Salmonella DNA from 5*μ*g–500 fg. (c) The qLAMP method. (D) The qPCR method.

To ascertain the detection limit of all four methods, 10-fold serial dilution of DNA template was used to compare all four assays. The detection limits of the LAMP assay were 50 pg, as shown in Figure 2(a). For the PCR reaction, 198 bp amplicon was found in lanes 1, 2, and 3, as shown in Figure 2(b). Therefore, the sensitivity of the PCR method was 50 ng. For the qPCR method, the sensitivity was 5 ng as shown in Figure 2(d). For the qLAMP method, the detection results are shown in Figure 2(c).

From Figure 2, the sensitivity of PCR, qPCR, LAMP, and qLAMP was 50 ng, 5 ng, 50 pg, and 500 fg, respectively.

### 3.3. The Detection of Salmonella in Spiked and Naturally Contaminated Chicken Samples

Within 2 hr. of the enrichment time, the LAMP, PCR, qLAMP, and qPCR methods had detected *Salmonella* DNA in the spiked chicken. A total of 30 chicken samples from 10 markets around Pathum Thani province were enriched for 2 hr. After the DNA extraction and amplification, only eight samples were positively detected by the conventional PCR method. The LAMP, qLAMP, and qPCR methods similarly detected *Salmonella* in all 30 chicken samples as shown in Table 2.

## 4. Discussion

The *Salmonella* invA gene was chosen for molecular detection with the PCR and qPCR methods because it has a broad specificity for more than 100 *Salmonella* serovars while exhibiting excellent exclusivity for non-*Salmonella* strains [16, 17].

The invA gene detection using the LAMP method was more rapid and sensitivity than the PCR method. The reaction time of LAMP was about 50 min while the reaction time of PCR was about 2.5 hr. The reaction time of LAMP was about 40 min faster than PCR. The limitation of LAMP (50 pg) reaction was 1,000 times higher than PCR (50 ng). The PCR efficiency of detection was 10 times better in qPCR (5 ng).

When the qLAMP method was applied, the efficiency of the detection increased. 500 fg of *Salmonella* DNA was detected using the qLAMP method. The sensitivity was 100 times higher than by conventional LAMP. The 50 ng and 5 ng of DNA were found to be approximately equivalent to 5 × 10^10^ copies and 5 × 10^9^ copies of the invA gene [18]. As *Salmonella* invA are single-copy genes they were converted to cell numbers (1 gene copy = 1 cell) [18]. Therefore, 5 × 10^10^ copies and 5 × 10^9^ copies of invA gene were converted to 5 × 10^10^ cells and 5 × 10^9^ cells, respectively.

A single bacteria cell, which divides approximately every 30 minutes, can grow into a colony containing 10^7^–10^8^ cells [19]. So, it means that the 5 × 10^10^ cell of Salmonella is about 500 colonies or 500 CFU and the 5 × 10^9^ cells is about 50 colonies or 50 CFU of *Salmonella.* For the conventional LAMP and qLAMP methods, 5 and 5 × 10^−2^ CFU of *Salmonella* were detected. Chen et al. [13] also found that LAMP assay was more efficient than conventional PCR. Chen and his colleagues [3] use LAMP to detect Salmonella at 6.1 × 10^3^ CFU/g in spiked produce sample, whereas the limitation of conventional PCR for detecting Salmonella was 6.1 × 10^6^ CFU/g in spiked produce sample. So the sensitivity of PMA-LAMP was 10^3^ times higher than conventional PCR.

Moreover, Chen et al. [13] compared TaqMan qPCR and LAMP in detecting *Salmonella enterica* serovar Enteritidis. Their results showed that there was a detection limit of four copies per reaction using both assays.

Additionally, another LAMP assay for *Salmonella* detection that targeted the *phoP* gene was able to detect 35 CFU per reaction [20]. Srisawat and Panbangred [21] used the stn gene of *Salmonella* for LAMP amplification. The sensitivity was reported as 5 fg.

However, only a few studies have determined the qualitative capability of LAMP. One study investigating ammonia-oxidizing bacteria using LAMP found a good quantitative proficiency between 10^4^ and 10^10^ of DNA copies [22]. Another researcher investigated the quantitative capability of LAMP when combined with propidium monoazide sample treatment [14]; the detection limits were 3.4 to 34 viable Salmonella cells in pure culture and 6.1 × 10^3^ to 6.1 × 10^4^ CFU/g in spiked produce samples [14].

The LAMP, qLAMP, and qPCR methods detected *Salmonella* on all the chicken samples collected from the markets because the sensitivity of these methods is high. However, at least 100 colonies of *Salmonella* can cause the disease [23, 24]. Even though all the chicken samples were found to be infected with *Salmonella*, the colony number was very low. This quantity of *Salmonella* does not cause the disease in humans.

Therefore, both LAMP and qLAMP have more dominants than PCR and qPCR in many aspects. They have more specificity (both LAMP used 2 pairs of primer, while as PCR used only one pair of primer), were more rapid (both LAMP used only 45-60 min to operate while both PCR used 1.5-2 hr. to operate), have more sensitivity, and have less complexity (LAMP technique do not require the complicated equipment).

Nevertheless, the disadvantage of LAMP is the use of indirect detection methods such as the turbidity of magnesium pyrophosphate, electrophoresis, and SYBR Green dye, which cannot differentiate between target products and nonspecific products, thereby leading to false positive [25]. So Wei and colleagues [26] used a molecular beacon to avoid the false positive by producing the fluorescence signals only when the molecular beacon binds to the target DNA. Moreover, the self-quenching and dequenching fluorogenic probes called fluorescence of loop primer upon self-dequenching-LAMP (FLOS-LAMP) [27] can reduce the nonspecific detection or false positive in the LAMP technique.

And even though LAMP has more sensitivity than both PCR and qPCR, our results show that the limitation of qLAMP (500 fg of Salmonella DNA) was 100 times higher than conventional LAMP (50 pg of Salmonella DNA). In terms of speed, the qLAMP can detected target DNA faster than PCR, qPCR, and LAMP and may be less susceptible to inhibitors than qPCR [28, 29]. The qLAMP showed low false positive proportions than the magnesium pyrophosphate turbidimetric LAMP [30]. These reductions may be due to other factors besides amplification inhibitor [31].

## 5. Conclusion

This research found that PCR, LAMP, qLAMP, and qPCR were efficient methods of detecting *Salmonella* contamination in chicken. Especially, since both LAMP and qLAMP are more rapid, reliable, and robust for *Salmonella* detection in chicken samples and may be a valuable tool for routine testing. In addition, the qLAMP method is the most efficient in terms of sensitivity and rapidity.

## Figures and Tables

**Table 1:** Primer sequences used for LAMP and PCR amplification.

Name	Sequence (5′-3′)	Length (bp)	Position^a^
F3	CGGCCCGATTTTCTCGG	17	503-520
B3	CGGCAATAGCGTCACCT	17	665-682
FIP	GCGCGGCATCCGCATCAATA-TGCCCGGTAAACAGATGAGT	40	573-592 (F1c) 527-546 (F2)
BIP	GCGAACGGCGAAGCGTACTG-TCGCACCGTCAAAGGAAC	38	593-612 (B1c) 635-652 (B2)

^a^The positions are numbered based on the coding sequence of the *Salmonella invA* gene (GenBank: M90846). F3 and B3 were used in PCR and qPCR reaction.

**Table 2:** The number of Salmonella infected sample from 10 market by PCR, qPCR, LAMP, and qLAMP method.

Method	PCR	qPCR	LAMP	qLAMP
Positive samples	8/30	30/30	30/30	30/30

## Data Availability

The data used to support the findings of this study are available from the corresponding author upon request.
